# Infant HIV diagnosis and turn-around time for testing in Malawi, 2015

**DOI:** 10.4102/ajlm.v9i1.904

**Published:** 2020-11-26

**Authors:** Hammad Ali, Peter Minchella, Geoffrey Chipungu, Evelyn Kim, James Kandulu, Dalitso Midiani, Andrea Kim, Mahesh Swaminathan, Steve Gutreuter, John Nkengasong, Daniel Singer

**Affiliations:** 1 Division of Global HIV and Tuberculosis, Centers for Disease Control and Prevention, Atlanta, Georgia, United States; 2 Division of Global HIV and Tuberculosis, Centers for Disease Control and Prevention, Lilongwe, Malawi; 3 Ministry of Health, Lilongwe, Malawi

**Keywords:** HIV, Malawi, infant HIV testing, turn-around time

## Abstract

**Background:**

For HIV-exposed infants in Malawi, there are missed opportunities at each step of the testing and treatment cascade.

**Objective:**

This study assessed factors associated with HIV positivity among infants in Malawi and turn-around times for infant HIV testing.

**Methods:**

HIV testing data for infants aged 0–18 months from 2012 to 2015 were extracted from the Malawi HIV laboratory information management system and analysed using logistic regression. Turn-around time was defined as time between collection of samples to results dispatch from the laboratory.

**Results:**

A total of 106 997 tests were included in the analyses. A subset of 76 006 observations with complete dates were included in the turn-around time analysis. Overall positivity was 4.2%. Factors associated with positivity were increasing age (infants aged 3–6 months: adjusted odds ratio [aOR] = 2.24; infants aged 6–9 months: aOR = 3.42; infants aged > 9 months: aOR = 4.24), female sex (aOR = 1.08) and whether the mother was alive and not on antiretroviral therapy at time of the infant’s test (aOR = 1.57). Provision of HIV prophylaxis to the infant after birth (aOR = 0.38) was found to be protective against HIV positivity. The median turn-around time was 24 days (increased from 19 to 34 days between 2012 and 2015).

**Conclusion:**

Infant HIV positivity has decreased in Malawi, whereas turn-around time has increased. Factors associated with positivity include increasing age, female sex, and whether the mother was alive and not on antiretroviral therapy at the time of the infant’s test.

## Introduction

Malawi has a high prevalence of HIV; in 2016, 9.2% of persons aged 15–49 years, 920 000 people (550 000 women, 60%) aged 15 years and older, and 110 000 children (0–14 years of age) were living with HIV.^1^ Among all people living with HIV, 680 000 were on antiretroviral therapy (ART) as of 2016.^1^ Currently, there are 717 sites that offer ART to HIV-positive patients in Malawi and there are nine laboratories nationwide that provide infant HIV virologic testing.^2^ Like many countries in sub-Saharan Africa, Malawi is striving towards achieving the Joint United Nations Programme on HIV/AIDS 90-90-90 targets. These targets include 90% of people living with HIV diagnosed, 90% of the people living with HIV with known status accessing treatment, and 90% of those who are on treatment virally suppressed.^3^ In Malawi, an estimated (antiretroviral adjusted) 76.8% of 15–64-year-old people living with HIV know their HIV status, 91.4% who know their status self-report current use of ART and 91.3% of those who self-report ART use were virally suppressed.^4^ The second 90 requires ‘Test and Start’, which is defined as provision of ART for all people living with HIV irrespective of their cluster of differentiation 4 counts.^5^

National guidelines recommend that HIV DNA should be detected using polymerase chain reaction (PCR) in infants who are exposed to HIV. When resources and facilities are available, this should happen at the first opportunity after the infant is older than 6 weeks.^6^ Blood is collected by facility nurses and dried blood spot specimens are created and sent to one of the nine laboratories nationwide that provide infant HIV virologic testing. If the PCR test is negative, a rapid antibody test is done from age 12 months onwards. If the rapid antibody test done at age 12 months returns negative, another rapid antibody test is done at age 24 months, if breastfeeding stopped at least 6 weeks before. If still breastfeeding, then the test is done six weeks after stopping breastfeeding.

In spite of these clear guidelines, HIV diagnosis in HIV-exposed infants has remained a challenge in the country. The Malawi Population-Based HIV Impact Assessment 2015–2016 showed that around half (49.4%) of infants born to HIV-positive women received a virologic HIV test within 2 months of birth, and 29.3% received a test between 2 and 12 months.^4^ These Malawi Population-Based HIV Impact Assessment estimates are based on a self-report of the mother and are higher than the estimates from a 2014 cohort study, which showed that only 39% – 41% of HIV-exposed infants older than age 24 months had documented evidence of an HIV test result, with 3% of those tested being positive.^2^ About 82% – 89% of those who had a positive result or who were diagnosed with severe HIV disease were initiated on ART.^2^ These data highlight the potential opportunities missed at each step of the testing and treatment cascade for HIV-exposed infants, that is, more infants need to be tested and 10% – 20% of those tested positive were not started on ART.

Malawi started universal treatment (i.e., ‘Test and Start’) for HIV-positive children younger than age 12 months in 2011.^7^ In 2014, this was scaled up to cover all children younger than age 5 years,^6^ and in 2016 Malawi implemented ‘Test and Start’ to cover all people living with HIV nationally.^8^ One component of ‘Test and Start’ implementation is scaling up of HIV virologic testing among infants born to HIV-positive mothers, and this requires a rapid expansion of laboratory services in Malawi.

There is a paucity of adequate paediatric HIV data generally in Malawi and specifically on factors associated with infant HIV positivity. In addition to difficulties in testing all infants born to HIV-positive mothers, there have been significant challenges in turn-around times for infant HIV test results, thus affecting early diagnosis in Malawi. Turn-around time is a metric used to assess the length of time between steps in the laboratory testing process. Longer turn-around times lead to delays in getting test results, which lead to delayed diagnoses. Any delay in diagnosis invariably leads to delays in the initiation of treatment, leading to poorer health outcomes that can result in higher mortality rates among untreated infants. The objectives of this study were to look at HIV positivity in infants in Malawi over time, and assess factors associated with positivity. In addition, we looked at the turn-around time for infant HIV testing to understand the timeliness of diagnoses.

## Methods

### Ethical considerations

This study was approved as exempt 45 CFR 46.101(b)(4) by the Centers for Disease Control and Prevention institutional review board (#6890) and the National Health Science Research Committee, Ministry of Health, Malawi (#1675).

No extra data were collected for the purpose of these analyses. The Malawi HIV laboratory information management system (LIMS) database does not include patient names but does include unique patient identification numbers, which were scrambled prior to analysis, so that they may be used to clean or de-duplicate the data set but cannot be linked back to a patient.

### Study design

A cross-sectional analysis was conducted on data from the LIMS. The United States President’s Emergency Plan for AIDS Relief supported the development of a LIMS in Malawi, which captures basic information on each infant HIV and viral load sample sent for testing. Although originally developed to project reagent needs for laboratories conducting virologic testing and thus keep track of laboratory functions, the database offers important information both on the utilisation of laboratory services as well as on the effectiveness of HIV services. Data included in the LIMS are routinely collected as part of regular provision of HIV services. These data include patient demographics, records of sample transportation and laboratory test outcomes. All data are entered into encrypted databases that are centrally stored and are password protected.

### Data analysis

Data between 2012 and 2015 were extracted from the infant HIV testing database of the Malawi LIMS. Records for infants aged 0 to 18 months at the time of specimen collection were included in the analysis. Tests were excluded from the analysis if the test result was missing. There were two patient identification numbers available in the system that were used. Data were excluded if both patient identification numbers were missing to avoid duplication. Based on the patient identification numbers, if there was more than one record found in the database for a patient identification number, the record with the latest test date was kept and the others dropped before analysis. Infant HIV positivity was defined as detection of HIV DNA using a PCR test. In Malawi, specimens with positive or indeterminate results are re-tested for confirmation. Table 1 shows the variables from the LIMS database that were included in the analysis. Overall turn-around time was defined as the time between collection of the sample at the healthcare facility to the dispatch of the results from the laboratory back to the facility. Data were dropped from the turn-around time analysis if the date values were implausible (e.g., if the date of the test was before the date of the collection or date of birth). Mean and median turn-around times were calculated between all major steps of the process.

**TABLE 1:** Variables extracted from the laboratory information management system to review infant HIV diagnosis and turn-around time for testing in Malawi, 2012–2015.

Category	Variables included
Demographics	Region, age, infant sex
Clinical information	Infant HIV registration number, HIV care clinic number, multiple-birth indicator, birth order, infant HIV prophylaxis at birth, infant ART continued (continuation of ART after completion of prophylaxis as empiric therapy), infant breast feeding status, whether mother was alive (on ART or not on ART) or deceased at the time of the infant test, mother ART status during pregnancy or labour, reason for PCR testing (e.g., routine vs severe disease)
Date of each step	Date sample drawn at the health facility, date sample received at lab, date sample tested, date result dispatched back to health facility
Test information	Test result

ART, antiretroviral therapy; PCR, polymerase chain reaction.

Since the Malawi LIMS includes routinely collected data, a number of variables had missing values. Observations were missing from the records for infant feeding option (60%), reason for PCR testing (37%), infant sex (26%), ART and life status of the mother (alive and on ART, alive but not on ART, deceased; 12%), multiple-birth indicator (8%), region (1.2%) and age (0.2%). We presumed that observations in all these variables were missing at random and used fully conditional chained equations^9^ to impute 50 completed data sets for analysis.

Bivariate and multivariable logistic regression analyses were undertaken on each of the multiply imputed data sets, and combined using Rubin’s rules,^10^ to identify factors associated with infant HIV positivity. The initial multivariable model included all variables approaching significance (*p* < 0.25) in the bivariate model, plus all plausible two-way interactions provisionally detected by changes in odds ratios between the bivariate analysis and a full main-effects model. We eliminated covariates one at a time – beginning with the interactions – based on *p*-values from Wald tests to identify the simplest plausible multivariable model, retaining only covariates and interactions for which *p* ≤ 0.05. Our inferences were based upon the adjusted odds ratios (aOR) from the final multivariable model and their 95% confidence intervals (CI). STATA version 15 (StataCorp LP, College Station, Texas, United States) was used to conduct analyses.

## Results

The 2012–2015 data set included 125 713 tests, and 106 997 tests (85.1%) from unique infants aged 0–18 months which were included in the analysis of associations with HIV positivity. Of those, only 76 006 tests had complete date recordings and those were used for the analysis of turn-around times. A total of 23 954 tests were conducted in 2012, 27 437 in 2013, 29 880 in 2014 and 25 726 in 2015. Most infants tested were younger than 3 months old (66.6%); there were almost equal numbers of male (39 628) and female (39 525) infants and most infants were from southern Malawi (69.1%; Table 2).

**TABLE 2:** Population characteristics and factors associated with a positive HIV result in infants in Malawi, 2012–2015.

Characteristics	Original (*N* = 106 997)				Multiple imputations			
	Tests		HIV-positive		Crude OR*	95% CI	Adjusted OR*	95% CI
	*n*	%	*n*	%				
**Region**								
Northern	6066	5.7	220	3.6	Ref.	-	-	-
Central	26 634	25.2	945	3.5	0.98	0.84–1.14	†	-
Southern	73 000	69.1	3222	4.4	**1.23**	**1.07-–1.41**	†	-
**Year**					0.82	0.79–0.84	0.93	0.90–0.96
**Age**								
< 3 months	71 127	66.6	1649	2.3	Ref.		Ref.	
3–6 months	21 093	19.7	1215	5.8	**2.57**	**2.38–2.77**	**2.24**	**2.07–2.42**
6–9 months	8902	8.3	856	9.6	**4.47**	**4.10–4.87**	**3.42**	**3.13–3.74**
> 9 months	5688	5.3	703	12.4	**5.92**	**5.40–6.50**	**4.24**	**3.84–4.67**
**Sex**								
Male	39 628	50.1	1701	4.3	Ref.		Ref.	
Female	39 525	49.9	1840	4.7	**1.07**	**1.00–1.14**	**1.08**	**1.01–1.16**
**Multiple births**								
Yes	799	0.8	17	2.1	Ref.	-	-	-
No	96 650	99.2	4120	4.3	**1.93**	**1.20–3.11**	-	-
**Birth order**								
0	106 329	99.4	4434	4.2	2.04	0.97–4.33	-	-
1	336	0.3	7	2.1	Ref.		-	-
2	327	0.3	7	2.1	1.03	0.36–2.96	-	-
3	5	0.0	0	0.0	‡		-	-
**Infant given HIV prophylaxis**								
No prophylaxis provided	3561	3.3	646	18.1	Ref.		Ref.	-
Prophylaxis provided	39 283	36.7	999	2.5	**0.12**	**0.11–0.13**	**0.38**	**0.33–0.44**
Status unknown	64 153	60.0	2803	4.4	**0.21**	**0.19–0.23**	**0.52**	**0.43–0.63**
**Infant ART continued**								
No ART provided	9713	9.1	772	7.9	Ref.		-	-
ART provided	30 173	28.2	768	2.5	**0.30**	**0.27–0.33**	-	-
Status unknown	67 111	62.7	2908	4.3	**0.52**	**0.48–0.57**	-	-
**Feeding options**								
Ongoing exclusive breastfeeding	37 661	88.6	1212	3.2	Ref.		-	-
Ongoing complementary feeding	1332	3.1	142	10.7	**2.93**	**2.52–3.41**	-	-
Ongoing mixed feeding	3081	7.2	303	9.8	**2.63**	**2.37–2.91**	-	-
Stopped breastfeeding < 6 weeks	163	0.4	15	9.2	**2.16**	**1.35–3.47**	-	-
Stopped breastfeeding > 6 weeks	266	0.6	26	9.8	**2.70**	**1.96–3.74**	-	-
**Mother’s ART and life status**								
Positive – alive on ART	12 363	13.1	288	2.3	Ref.	-	Ref.	-
Positive – alive not on ART	81 983	86.8	3733	4.6	**1.86**	**1.65–2.10**	**1.57**	**1.36–1.82**
Dead	72	0.1	4	5.6	2.28	0.83–6.28	1.67	0.60–4.65
**ART in pregnancy or labour**								
ART provided	38 583	36.1	983	2.5	Ref.	-	Ref.	-
No ART provided	2646	2.5	599	22.6	**11.19**	**10.02–12.51**	†	-
Status unknown	65 768	61.5	2866	4.4	**1.74**	**1.62–1.88**	†	-
**Reason for PCR**								
HIV-exposed infant	66 464	99.2	2437	3.7	Ref.	-	Ref.	-
HIV-exposed infant with presumed severe HIV disease	262	0.4	43	16.4	**3.25**	**2.34–4.52**	**2.37**	**1.65–3.39**
Unknown exposure, displaying symptoms	145	0.2	11	7.6	**2.09**	**1.21–3.61**	0.73	0.41–1.30
Other	149	0.2	11	7.4	**1.98**	**1.12–3.50**	1.00	0.55–1.81

ART, antiretroviral therapy; OR, odds ratio; PCR, polymerase chain reaction; Ref., reference category.

* , Bold odds ratio values are significant.

† , See interactions.

‡ , Could not be estimated.

### HIV positivity

The overall infant HIV positivity between 2012 and 2015 was 4.2% (*n* = 4448), which reduced from 5.7% in 2012 to 3.1% in 2015. Positivity was 4.3% in male infants (*n* = 1701) and 4.7% in female infants (*n* = 1840) and increased by age (2.3% in infants aged < 3 months old, 5.8% in infants aged 3–6 months old, 9.6% in infants aged 6–9 months old, and 12.4% in infants aged > 9 months old; Table 2). Positivity was higher if the mother was not on ART during pregnancy or labour (22.6%) or if the mother’s ART status was unknown (4.4%), compared to women who were on ART during pregnancy or labour (2.5%). Positivity was highest for infants of deceased mothers (5.6%), compared to those whose mothers were alive and on ART (2.3%). Positivity was higher if the infant was not given HIV prophylaxis at birth (18.1%) compared to those who were given prophylaxis at birth (2.5%).

On multivariable analyses, the odds of testing positive for HIV decreased by 18% per year (aOR 0.82, 95% CI: 0.70–0.84). Other factors significantly associated with testing positive for HIV were increasing age of the infant (3–6 months: aOR 2.24, 95% CI: 2.07–2.42; 6–9 months: aOR 3.42, 95% CI: 3.13–3.74; > 9 months: aOR 4.24, 95% CI: 3.84–4.67), female sex (aOR 1.08, 95% CI 1.01–1.16), whether the mother was alive but not on ART at the time of the infant HIV test (aOR 1.57, 95% CI: 1.36–1.82), and if PCR was conducted because the infant was presumed to have severe HIV disease (aOR 2.37, 95% CI: 1.65–3.39) (Table 2). Immediate postpartum provision of HIV prophylaxis to infants was protective against testing positive for HIV (aOR 0.38, 95% CI: 0.33–0.44). The effects of ART during pregnancy or labour varied among regions, as manifested by an interaction between region and ART provision during pregnancy or labour. Infants born to mothers who received ART during pregnancy or labour were equally likely to test positive regardless of region. Conversely, the aORs for infants born to mothers who did not receive ART during either pregnancy or labour varied among regions, and infants born in the central region had the lowest aOR (2.80, 95% CI 2.06–3.81) relative to baseline (infants born in the northern region to mothers who received ART during pregnancy or labour) (Table 3).

**TABLE 3:** Interaction between antiretroviral therapy in labour or pregnancy and region as factors associated with positive HIV results in infants in Malawi, 2012–2015.

ART in pregnancy or labour	Region	Adjusted odds ratio*	95% CI
ART provided	Northern	Ref.	-
	Central	0.96	0.74–1.24
	Southern	1.19	0.93–1.51
No ART provided	Northern	**4.07**	**2.27–6.09**
	Central	**2.80**	**2.06–3.81**
	Southern	**5.35**	**4.05–7.07**
Status unknown	Northern	**1.90**	**1.34–2.70**
	Central	**1.40**	**1.04–1.83**
	Southern	**1.47**	**1.10–1.95**

ART, antiretroviral therapy.

* , Bold values are significant.

### Turn-around time

The overall median turn-around time between HIV-related sample collection at the healthcare facility to the dispatch of results from the laboratory back to the facility was 24 days (interquartile range: 15–36 days), whereas the mean turn-around time was 29.1 days (standard deviation: 22.5 days) (Table 4). The median turn-around time was 19 days in 2012 but increased by 79% to 34 days in 2015. Median turn-around time was longest in the northern region (28 days, interquartile range: 20–40 days), followed by central (27 days, interquartile range: 17–40 days) and southern region (23 days, interquartile range: 15–35 days). Median times increased for all steps from 2012 to 2015: median turn-around time between sample drawn at the health facility and being received at the laboratory increased from 11 days in 2012 to 16 days in 2015; the median turn-around time between the sample being received and the sample testing increased from 4 to 6 days; median turn-around time between sample testing and the dispatch of the result from the laboratory back to the facility increased from 0 to 3 days.

**TABLE 4:** Mean and median turn-around time by year for HIV tests in infants in Malawi, 2012–2015.

Median turn-around time between		Mean	Standard deviation	Median (interquartile range)				
				Overall	2012	2013	2014	2015
Overall: Sample drawn at the facility to result dispatched from the laboratory		29.1	22.5	24 (15–36)	19 (12–31)	26 (16–37)	24 (18–31)	34 (18–51)
Step 1	Sample drawn at the facility and received at the laboratory	15.8	15.1	12 (7–20)	11 (7–19)	10 (6–18)	14 (9–18)	16 (8–31)
Step 2	Sample received and tested	9.8	10.6	6 (3–13)	4 (2–8)	9 (4–21)	5 (3–10)	6 (3–14)
Step 3	Sample tested and result dispatched from the laboratory	3.5	12.1	2 (0–3)	0 (0–1)	2 (1–2)	2 (2–4)	3 (2–6)

IQR, interquartile range.

## Discussion

This study used data from Malawi LIMS, a routinely collected HIV data source, and showed that the overall HIV positivity in HIV-exposed infants reduced from 5.7% in 2012 to 3.1% in 2015; however, the overall median turn-around time between collection of the sample at the healthcare facility to the dispatch of the results from the laboratory back to the facility increased from 19 days in 2012 to 34 days in 2015. In addition, LIMS data were analysed to identify factors associated with HIV positivity in infants, including infant age and maternal ART uptake. These results highlight the point that routine testing data from LIMS can be used not only to inform service delivery but to conduct epidemiological analyses to fill gaps in knowledge about HIV infection.

The positivity results from this study (3.1% in 2015) are similar to the results reported by the Ministry of Health (3% in 2014).^2^ Infant HIV positivity reduced between 2012 and 2015 and prevention of mother-to-child-transmission (PMTCT) initiatives are likely the main reason for this. Specifically, implementation of option B+ (where all pregnant women living with HIV are started on lifelong ART irrespective of their cluster of differentiation 4 count)^11^ – as opposed to option A (in which pregnant women receive ART prophylaxis antenatal and intrapartum, along with a postpartum ‘tail’ regimen) and option B (in which all women receive ART starting in the antenatal period with continued treatment throughout the breastfeeding duration) – has reduced the mother-to-child-transmission (MTCT) of HIV. The Malawi Population-Based HIV Impact Assessment found that 97.9% of self-reported HIV-positive women aged 15–49 years who gave birth in the last 12 months before the survey reported having received ART.^4^ It is important to note that Malawi introduced option B+ in 2011, before it became a recommendation by the World Health Organization.^12^ Since then, the coverage of pregnant women who received ART to prevent MTCT increased from 62% in 2012 to 79% in 2015 (and further increased to > 95% in 2018).^13^ Owing to the increase in the number of women who received ART to prevent MTCT, the number of new HIV infections averted have increased, from 8100 in 2012 to 8600 in 2015 (and further increased to 9600 in 2018), and the number of new infections among children aged 0–14 years has decreased from 9300 in 2012 to 6400 in 2015 (and further decreased to 3500 in 2018).^13^ In addition to the increase in PMTCT, the proportion of infants born to HIV-positive women who received an HIV virological test within the first 2 months after birth increased from 5% in 2012 to 22% in 2015 (and further increased to > 95% in 2018).^13^ Thus, the reduction in positivity rates is probably led by the reducing MTCT rates due to option B+. Another possible factor contributing to decreased positivity is the increase in testing in healthy infants due to the expansion of early infant diagnosis testing.

This study identified a number of factors found to be associated with HIV positivity in infants. Increasing age was associated with the risk of testing positive. This could be due to a delay in infant testing, duration of breastfeeding, or lack of access or adherence to care and treatment. Most mothers breastfeed in Malawi and the longer a mother who is not on ART breastfeeds, the greater the risk of transmission of HIV to the infant.^14^ Current Malawi guidelines dictate that HIV-exposed infants should be tested between age 6 and 8 weeks. However, this finding shows that at least some of the infants are not getting tested by 6–8 weeks which may be because mothers are not accessing care and treatment for their HIV. This could be due to non-disclosure issues which can pose a risk for sexual transmission of HIV to the partner, and impact the implementation of PMTCT.^15^ Also, since the latest test result was selected from the LIMS for this study, some results may reflect a repeat or confirmatory test and thus could have contributed to the observed late testing.

Infants born to mothers who received ART during pregnancy or labour experienced indistinguishable risks of HIV infection across all regions. However, infants born to mothers who did not receive ART during either pregnancy or labour were more likely to have been infected, and their odds ratio varied among regions. Infants born in the central region to mothers who did not receive ART had a slightly lower aOR relative to baseline than their counterparts born in the northern and southern regions; however, the confidence intervals overlapped for all three regions. Infants were at higher risk of being tested positive if mothers were alive and not on ART at the time of the test compared to those whose mothers were alive and on ART. This shows the importance of providing ART during pregnancy to women living with HIV^14,16,17,18,19^ and during labour,^18^ which is well established as a PMTCT measure.

Human immunodeficiency virus-exposed infants not provided with HIV prophylaxis at the time of birth were at an increased risk of being HIV-positive as opposed to those who were provided with prophylaxis at birth. In addition, HIV-exposed infants with presumed severe HIV disease at The time of the test were at a higher risk of being HIV-positive. Various studies have reported that suboptimal use of ART and inadequate skilled care during the delivery are factors that increase MTCT.^14,16^ Human immunodeficiency virus prophylaxis reduces the concentration of HIV virus in the infants’ blood.^20,21^ Thus, HIV prophylaxis should be provided to all exposed infants to reduce perinatal transmission of HIV and those who test positive should be started on ART immediately. Lastly, female infants were at a higher risk of testing HIV-positive compared to male infants. There is some evidence that female infants, compared to male infants, have higher susceptibility to HIV infection before birth. One study reported a significant association between sex and HIV infection, with female infants being at a much higher risk for HIV acquisition than male infants at birth, suggesting in utero acquisition.^22^

Currently, infant HIV virologic testing in Malawi depends on the availability of equipment, laboratory space and trained technicians.^23,24^ Out of the 28 districts in Malawi, only 7 have molecular laboratories that can perform infant HIV virologic testing. Therefore, samples need to be transported to these laboratories for testing from the districts with no molecular laboratories. This can increase the turn-around time for testing, resulting in delays in the return of the result back to the patient and start of treatment if the result is HIV-positive. Multiple factors can prolong turn-around time, including delays with sample transport due to inclement weather, shortage of fuel and holidays.^25^ Laboratory factors can also delay turn-around time, including staff shortages, work overload, stock-out of reagents, issues with equipment and other administrative delays.^26^ The increase in turn-around time documented in this study occurred during a period of rapid expansion of option B+ for PMTCT. The PMTCT programme uses the same mechanisms for transport of samples and ART and the same laboratory facilities for HIV viral load testing. The increased turn-around time likely represents stresses on the system across all levels as scale-up occurred. A sample tracking and online result reporting system – so samples can be tracked during delivery and results can arrive at the facility as soon as tested – can help with improving turn-around time and providing results as soon as possible to clinicians. Another option could be the use of HIV point-of-care testing which can deliver results at the test site. Although point-of-care testing is currently not available on a routine basis in Malawi, a point-of-care early infant diagnosis implementation pilot conducted in Malawi reduced the turn-around time from collection of sample to ART initiation and increased the proportion of patients initiating ART.^27^

The findings in this study are strengthened by the fact that the data set used in this study is the national laboratory data set which includes data from all tests conducted at the national laboratories in the country. This includes tests conducted at the public health facilities and the externally managed facilities and there is a minimal chance of testing happening at a laboratory outside the national laboratories. Additionally, the data are available from 2012 onwards which allows for interpretation of the trends over time. The availability of turn-around time data provides a rare opportunity to monitor the delivery of laboratory services on a national scale. Findings from this study may be applicable throughout sub-Saharan Africa, especially in countries that are scaling up virologic testing.

### Limitations

Like all studies, this study has limitations. Firstly, as the data set only included routinely collected variables, only a limited set of variables could be included in the logistic regression. Some other important variables that could provide insight into other demographic, behavioural and clinical factors associated with HIV positivity in infants could not be included, as they are not collected routinely. Secondly, multiple patient identification numbers were available in the data set. However, none of the identification numbers were completely available for the majority of the patients. Therefore, the data had to be de-duplicated using various patient identification numbers, which, in spite of all efforts, may have led to some duplication in the data set. Thirdly, a number of variables had missing data. This was due to incomplete data recording and changes in the HIV test requisition form over time. We attempted to address this by doing multiple imputations and using the imputed data to conduct the regression analyses. For the turn-around time analysis, nearly 40% of tests had to be excluded from analysis, if the dates were missing or if the dates were implausible. Better data management and quality control is required to resolve issues of missing data, which will improve the measurement of indicators. Fourthly, the variable ‘mother’s life and ART status’ at the time of the infant’s HIV test may not have been correctly entered. It seems that when the mother’s life and ART status were missing, it was recorded as mother not on ART. This is important to note, because in the option B+ era in Malawi, it is unlikely that so many women were HIV-positive and not on ART. Lastly, LIMS is not connected to the clinical systems; thus, we were unable to ascertain outcomes of positive tests, that is, what proportion of infants were put on treatment and how quickly.

As LIMS is a routinely collected data source, it can potentially be used for surveillance purposes. However, as described, currently there are a number of data quality and completeness issues that may limit the ability of LIMS to be used as a surveillance system. Attention to detail is required to improve the quality and completeness of LIMS data, and routine data cleaning and validation procedures should be implemented before LIMS can be considered as a surveillance system. In addition, a formal comparison of data generated by LIMS and those generated by other data systems (e.g., electronic medical records) is required to validate the use of LIMS as a surveillance system in Malawi.

### Conclusion

Although infant HIV positivity has decreased over time in Malawi, the turn-around time has increased over the same period of time. Active engagement from all stakeholders is required to reduce the turn-around time to assist with implementation of ‘Test and Start’ among infants in the country. The earlier HIV is detected in infants, and treatment started by the clinician, the better the outcomes among these infants. Therefore, it is imperative to test early and provide the results to clinicians as soon as possible so that appropriate care and treatment are provided to infants. Although this study shows that LIMS can provide important information about infant HIV virologic testing, we do not know what proportion of patients actually received these results and what proportion of results were actually acted upon by clinicians. Although LIMS in Malawi was designed to provide information for the laboratories, it can provide other important epidemiological pieces of information. This evidence-based information can guide development and restructuring of policy and programmes in the country.
